# Enhanced Diffraction and Spectroscopic Insight into Layer-Structured Bi_6_Fe_2_Ti_3_O_18_ Ceramics

**DOI:** 10.3390/ma18153690

**Published:** 2025-08-06

**Authors:** Zbigniew Pędzich, Agata Lisińska-Czekaj, Dionizy Czekaj, Agnieszka Wojteczko, Barbara Garbarz-Glos

**Affiliations:** 1 Faculty of Materials Science and Ceramics, Department of Ceramics and Refractories, AGH University of Krakow, 30-059 Krakow, Poland; agdudek@agh.edu.pl; 2 Faculty of Mechanical Engineering and Ship Technology, Gdansk University of Technology, 11/12, Narutowicza St., 80-233 Gdansk, Poland; agata.czekaj@pg.edu.pl (A.L.-C.); dionizy.czekaj@pg.edu.pl (D.C.); 3 Department of Social Research, Forensic Methods and Physics, University of the National Education Comission, 2, Podchorążych St., 30-084 Kraków, Poland; barbara.garbarz-glos@uken.krakow.pl

**Keywords:** electron backscatter diffraction (EBSD), X-ray diffraction method, dielectric spectroscopy, layer-structured electroceramics

## Abstract

Bi_6_Fe_2_Ti_3_O_18_ (BFTO) ceramics were synthesized via a solid-state reaction route using stoichiometric amounts of Bi_2_O_3_, TiO_2_, and Fe_2_O_3_ powders. A thermal analysis of the powder mixture was conducted to optimize the heat treatment parameters. Energy-dispersive X-ray spectroscopy (EDS) confirmed the conservation of the chemical composition following calcination. Final densification was achieved through hot pressing. The crystal structure of the sintered samples, examined via X-ray diffraction at room temperature, revealed a tetragonal symmetry for BFTO ceramics sintered at 850 °C. Electron backscatter diffraction (EBSD) provided detailed insight into the crystallographic orientation and microstructure. Broadband dielectric spectroscopy (BBDS) was employed to investigate the dielectric response of BFTO ceramics over a frequency range of 10 mHz to 10 MHz and a temperature range of −30 °C to +200 °C. The temperature dependence of the relative permittivity (ε_r_) and dielectric loss tangent (tan δ) were measured within a frequency range of 100 kHz to 900 kHz and a temperature range of 25 °C to 570 °C. The impedance data obtained from the BBDS measurements were validated using the Kramers–Kronig test and modeled using the Kohlrausch–Williams–Watts (KWW) function. The stretching parameter (*β*) ranged from ~0.72 to 0.82 in the impedance formalism within the temperature range from 200 °C to 20 °C.

## 1. Introduction

Bismuth titanate (Bi_4_Ti_3_O_12_), a bismuth-layered structured ferroelectric material first discovered by B. Aurivillius [1,2,3], has been extensively used in electronics, particularly in capacitors, transducers, non-volatile memory devices, and high-temperature piezoelectric sensors. Enhancing its properties through modification with bismuth ferrite (BiFeO_3_) offers promising new functionalities. By combining these two compounds—each with distinct physical properties—novel materials with an enhanced multifunctionality can be developed. As reported in previous (now classical) studies [4,5], materials in the Bi_4_Ti_3_O_12_–BiFeO_3_ system exhibit ferroelectric, semiconducting, and ferromagnetic behaviors, making them attractive candidates for next-generation information processing and storage technologies. These Bi-containing Aurivillius-type oxides typically adopt a layered perovskite-related structure and conform to the following general formula: Bi_m+1_Fe_m−3_Ti_3_O_3m+3_. Their unique structure, composed of alternating bismuth oxide and perovskite-like layers, has garnered increasing interest in recent years.

**Figure 1:**
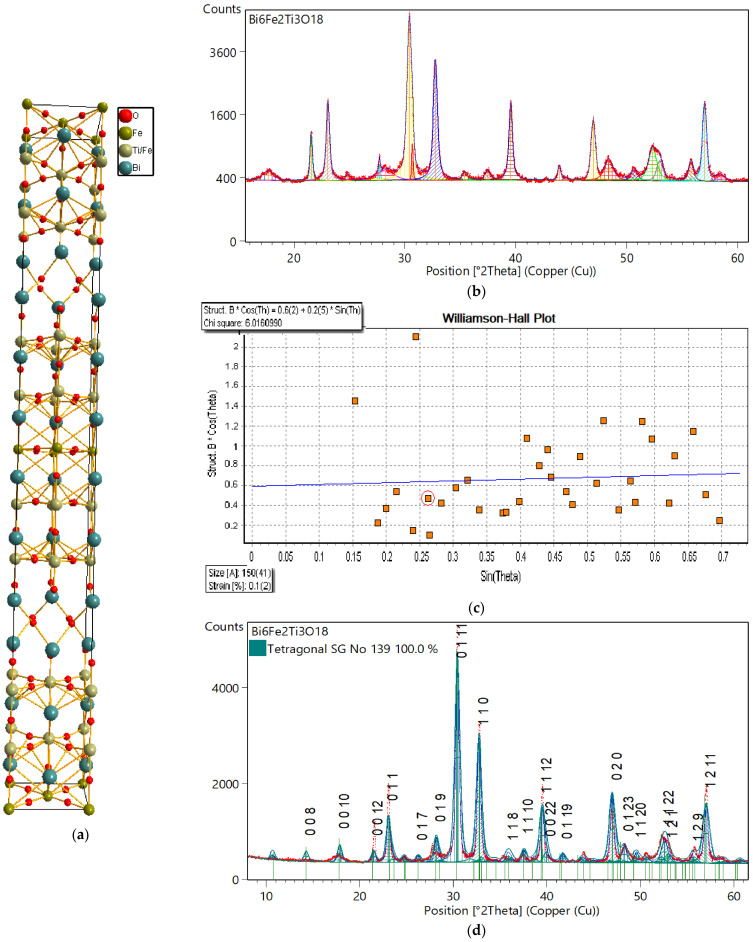
Ball-and-stick presentation of the unit cell of the compound with the Aurivillius structure—(**a**). Results of the line profile analysis of the X-ray diffraction pattern of Bi_6_Fe_2_Ti_3_O_18_ ceramics—(**b**). Williamson–Hall plot—(**c**). Refined structural analysis. The minimum intensity of the diffraction line, which was labeled with Miller indices, was 3%—(**d**).

Aurivillius [1,2,3] identified this family of oxides, characterized by alternating layers of (Bi_2_O_2_)^2+^ and (A_m−1_B_m_O_3m−1_)^2−^ stacked along the pseudotetragonal *c*-axis. The A-site can accommodate mono-, di-, or trivalent ions, or their mixtures, while the B-site includes Ti^4+^, Nb^5+^, Mo^6+^, W^6+^, and Fe^3+^. In the perovskite blocks, B-site ions are surrounded by oxygen octahedra that form linear O–B–O chains, with A-site ions fitting into the framework created by the BO_6_ octahedra. These perovskite slabs consist of *m* octahedral layers, where *m* can be an integer or a fractional value. Adjacent layers are offset by a_0_/√2 in the [110] direction (where a_0_ is the lattice constant of the ideal perovskite cell). The unit cell of the compound with the Aurivillius structure and *m* = 5 perovskite-like layers per slab is shown in Figure 1a.

Structural symmetry imposes constraints on polarization behavior depending on *m* [6,7]. When *m* is even, polarization is confined to the *a*–*b* plane; however, with an odd number of layers, a component of polarization can develop along the *c*-axis, nearly perpendicular to the layering. This has significant implications for device applications, as fewer allowed directions for spontaneous polarization often result in lower remanent polarization in many film orientations. Nevertheless, Aurivillius-type bismuth-based titanium oxide (Bi_4_Ti_3_O_12_) remains of great interest for integrated circuit memory and high-temperature piezoelectric sensor applications, exhibiting excellent electrical and electro-optic properties [8]. The compositional and structural tunability of bismuth oxide-layered perovskites—through ionic substitution and octahedral distortions—allows for fine control over their electrical behavior [9]. The ferroelectricity in these structures is closely linked to distortions in the coordination polyhedra of one or more cations.

This study focuses on the promising Aurivillius-phase material Bi_6_Fe_2_Ti_3_O_18_ (BFTO), chosen for its high dielectric transition temperature (Curie temperature *T* = 805 °C) and its second-order magnetoelectric effect [10,11,12]. The aim was to synthesize BFTO ceramics via the mixed oxide method and conduct a detailed structural and spectroscopic characterization. The compound fits the general formula Bi_m+1_Fe_m−3_Ti_3_O_3m+3_ with *m* = 5, corresponding to five perovskite-like layers per slab. The main investigative tools employed were electron backscatter diffraction (EBSD) and broadband dielectric spectroscopy (BBDS) [13].

Due to their high structural flexibility and the ability to exhibit both electric dipole and magnetic ordering at room temperature, Aurivillius-phase materials are compelling candidates for single-phase, room-temperature multiferroics. Recent reviews (e.g., [14]) have explored the characteristics of these layered structures and advances in their synthesis, yet BFTO has received comparatively little attention. To our knowledge, no EBSD investigations of BFTO have been reported.

EBSD enables a detailed analysis of crystalline orientation and structure. A stationary electron beam interacts with a tilted crystal sample, producing a diffraction pattern characteristic of the local crystal structure [15]. These patterns can reveal phase distributions, grain boundaries, and crystal quality. Proper sample preparation—particularly a deformation-free surface—is essential for obtaining high-quality EBSD patterns. By scanning the beam over a grid across a polycrystalline sample, orientation maps can be constructed, providing insight into the grain morphology and texture. This enables a comprehensive, quantitative description of the material’s microstructure.

In addition to EBSD, this study utilized a frequency-domain representation of the Kohlrausch–Williams–Watts (KWW) function [16,17,18] to analyze electrical relaxation. Immittance spectra were evaluated using complex impedance [19,20] providing a detailed view of the dielectric response.

## 2. Materials and Methods

Ceramic samples of Bi_6_Fe_2_Ti_3_O_18_ were synthesized via the conventional solid-state reaction method. Stoichiometric amounts of high-purity (99.9%, Aldrich Chemicals Co., Milwaukee, WI, USA) reagent-grade oxides—Bi_2_O_3_, Fe_2_O_3_, and TiO_2_—were accurately weighed and thoroughly mixed using an agate mortar and pestle to ensure homogeneous blending. The solid-state reaction proceeded according to the following reaction: 3Bi_2_O_3_ + Fe_2_O_3_ + 3TiO_2_→2Bi_6_Fe_2_Ti_3_O_18_.

A thermal analysis of the powder mixture was conducted to optimize the heat treatment parameters. It was found from the experimental data that layer-structured Aurivillius phases in the BFTO system appear at 600 °C [21] and they form within the temperature range of 650–700 °C [13].

According to the suggested scheme for the formation of Aurivillius phases in the Bi_2_O_3_–TiO_2_–Fe_2_O_3_ system, the formation of the Bi_m+1_Fe_m−3_Ti_3_O_3m+3_ compound is considered a multistage process [21,22]. In the early stages, compounds with smaller numbers of perovskite-like layers in the structure and BiFeO_3_ are formed. At the next stage, perovskite-like BiFeO_3_ is incorporated into a perovskite-like layer of the Aurivillius phases to increase the thickness of this layer until an Aurivillius phase of a given composition is formed.

The powder mixture was compacted into pellets at a uniaxial pressure of 300 MPa using a stainless-steel die (10 mm in diameter), yielding disc-shaped green bodies of approximately 10 mm in diameter and 1 mm thick. These compacts were placed in an alumina crucible and calcined in air at 750 °C for 10 h to promote the formation of the desired phase. Final densification was achieved via hot pressing at 850 °C for 3 h under a pressure of 20 MPa.

The crystalline structure of the sintered ceramics was analyzed at room temperature using X-ray diffraction (XRD) with a PANalytical X’Pert Pro diffractometer (PANalytical B.V., Almelo, The Netherlands) operating in θ–2θ geometry and employing Cu Kα radiation. Data were collected over a 2θ range of 8.0042–89.9962° with a continuous scan and a step size of Δ2θ = 0.008°. The diffraction patterns were evaluated using the X’Pert HighScore Plus (PANalytical B.V., Almelo, The Netherlands) [23] and Match! (Crystal Impact) software packages (Version 3.6.2.121) [24]. The structural identification was supported by the latest entries from the COD/AMCSD [25], ICSD [26], and ICDD [27] crystallographic databases.

The analysis method used in the present study for calculating the mean size and strain within the Bi_6_Fe_2_Ti_3_O_18_ oxide ceramics from the diffraction pattern is attributed to G.K. Williamson and W.H. Hall [28]. The Williamson–Hall method makes several assumptions. It simplifies the analysis by assuming that the size-broadening and strain-broadening contributions to the diffraction pattern can be combined not by convolution, but by a simple sum or sum of squares. While the effects of convolution are not always intuitively obvious, and some functions convolute in straightforward ways (e.g., two Gaussian or two Lorentzian functions), other functions may convolute more complexly. Despite these complexities, the Williamson–Hall method can be useful if applied relatively.

Microstructural and compositional analyses were carried out using a Thermo Scientific Apreo 2S field emission scanning electron microscope (SEM) (Waltham, MA, USA) equipped with an EDAX Octane Elect energy-dispersive X-ray spectroscopy detector (EDS, EDAX, Ametek, Berwyn, PA, USA) on a carbon-coated surface. The sample was mechanically polished (finished with colloidal silica) to achieve a scratch- and deformation-free surface suitable for EBSD measurements.

Electron backscatter diffraction (EBSD) was employed to examine the microstructural and crystallographic orientation of the samples. In EBSD, a stationary electron beam interacts with a tilted crystalline surface, generating Kikuchi patterns that are characteristic of the local crystal structure and orientation. These patterns are used to determine grain orientation, differentiate between phases, assess grain boundaries, and evaluate local crystalline quality. EBSD measurements were performed using an Apreo 2S (Thermo Scientific) scanning electron microscope (SEM) equipped with an EDAX Velocity EBSD camera. Data were collected at an accelerating voltage of 15 kV and a step size of 0.03 μm using the APEX EBSD software (Version 3.0601). The acquired EBSD data were processed using the EDAX OIM Analysis™ software (8.6.104x64), providing an advanced interpretation and visualization.

For dielectric measurements, the sintered BFTO ceramic discs were polished on both sides and coated with silver paste to form parallel-plate metal–insulator–metal (MIM) capacitor structures. Impedance spectroscopy (IS) measurements [20,29] were carried out over a frequency range from 100 mHz to 10 MHz and a temperature range from −120 °C to +200 °C. The measurements were conducted using the Alpha-A High-Performance Frequency Analyzer (Novocontrol Technologies GmbH & Co. KG, Montabaur, Germany) coupled with a Quatro Cryosystem for precise temperature control. Prior to the measurements, the system was cooled using liquid nitrogen. An AC perturbation voltage of 10 mV was applied, and impedance spectra were recorded during the heating cycle. To ensure thermal equilibrium, the data were acquired 15 min after each target temperature was reached. The temperature increment between measurements was ΔT = 5 °C. The WinDETA v3.9 software (Novocontrol) was used for data acquisition, visualization, and processing. To verify the reliability of the impedance data, the Kramers–Kronig test was applied using the B. Boukamp software (K-K Test V 1.01) [30]. This served as a consistency check between the real and imaginary components of the spectra.

Modeling of the experimental impedance data was conducted using both mathematical functions [18] and equivalent-circuit models. A key objective was to achieve a close agreement between the measured and modeled data with a minimal number of fitting parameters, acknowledging that some parameters may lack direct physicochemical meaning. The analysis was performed in the complex impedance formalisms. Nyquist plots (complex impedance diagrams) were fitted using the circular fitting method, while the imaginary components of impedance were modeled using the modified Kohlrausch–Williams–Watts (KWW) function [16,17]. Furthermore, the impedance behavior was interpreted using electrical equivalent circuits.

## 3. Results and Discussion

### 3.1. X-Ray Phase and Structural Analysis

The crystal structure of the powdered ceramics with a Bi_6_Fe_2_Ti_3_O_18_ composition were studied by the X-ray diffraction method at room temperature. A line profile analysis (LPA) was used to determine the microstructural parameters [31]. For this purpose, the X-ray diffraction pattern, shown in Figure 1b, was analyzed with the assumption of the pseudo-Voigt profile function. The following parameters of fitting were achieved: R_expected_ = 4.44%; R_profile_= 3.25%, R_weighted profile_ = 4.20%, and GOF = 0.947. The quality of the fitting procedure, as evidenced by R-parameters, was acceptably good, especially considering the complex system studied.

To retrieve microstructural parameters such as the average crystallite size and the microstrain, the simplified Williamson–Hall analysis was used [28]. The Williamson–Hall method demonstrates that the approximate formulas for the size broadening and strain broadening of the diffraction peak profile vary differently with respect to the Bragg angle, θ. The formula for size broadening varies as (cosθ)^−1^, whereas the formula for strain broadening varies as ~tanθ [32]. By plotting the product of the total broadening (of the diffraction line) and the cosine of the Bragg angle θ as a function of sinθ, the average strain component is obtained from the slope of the straight line (calculated by linear regression) and the average crystallite size component is obtained from the intersection of the straight line with the ordinate axis [31]. The relevant Williamson–Hall plot is given in Figure 1c. According to the performed LPA, it was found that the average crystallite size was 150 Å and the average crystallite (rms) strain was 0.1%.

The qualitative phase analysis of the X-ray diffraction pattern of Bi_6_Fe_2_Ti_3_O_18_ ceramics was performed with the Match! (Crystal Impact) software [24]. The analysis showed that, apart from the expected Bi_6_Fe_2_Ti_3_O_18_ phase, there are diffraction peaks that could be assigned to bismuth-based layer-structured Aurivillius phases with the number of layers in the slab other than *m* = 5. The results are shown in Table 1.

One can see from the Table 1 that the Aurivillius phases retrieved from the X-ray data bases exhibited an orthorhombic structure with the following space group numbers: 35 and 42. At the same time, it should be noted that the orthorhombic distortion of the tetragonal unit cell (i.e., the ratio of the base edge lengths of the cuboidal unit cell) characteristic for the Aurivillius phases included in Table 1 was close to 1. The figure of merit (FoM) is also shown in Table 1.

It is worth noting that the parent structure for a compound exhibiting *m* = 5 perovskite-like layers described by Aurivillius [33] was found to adopt the tetragonal structure (*I4/mmm*, Space Group No. 139). Due to the fact that Aurivillius phases exhibit small amounts of orthorhombic distortion, this structure was adopted to search for the unit cell of the Bi_6_Fe_2_Ti_3_O_18_ compound and its further refinement. The results are shown in Figure 1d. The following lattice parameters were obtained: *a*_0_ = *b*_0_ = 3.8625Å, *c*_0_ = 49.585Å. The refined average crystallite size was 156Å and the average crystallite (rms) strain was 0.41%. The parameters of the Rietveld refinement were as follows: R_expected_ = 4.47%; R_profile_= 9.45%, R_weighted profile_ = 12.02%, and GOF = 8.49.

### 3.2. EBSD Studies of Ceramics

**Figure 2:**
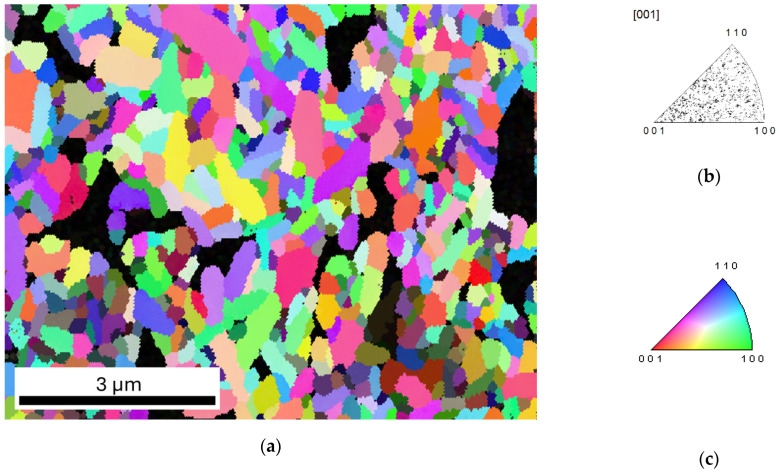
Inverse pole figure for Bi_6_Fe_2_Ti_3_O_18_ ceramics: (**a**) crystallographic orientation map; (**b**) inverse pole figure (IPF) showing the crystallographic orientation distribution of grains relative to the sample normal direction [001]; and (**c**) color-coded legend for IPF map.

**Figure 3:**
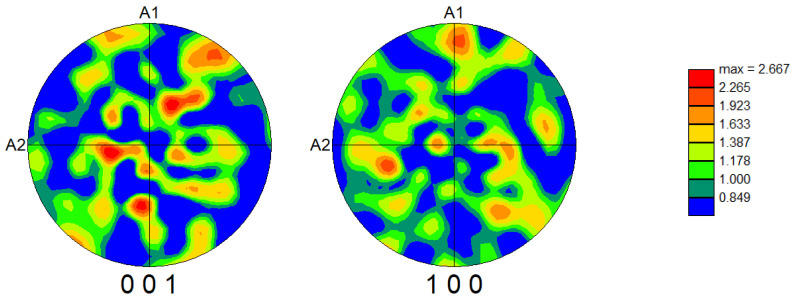
PF (pole figure) for Bi_6_Fe_2_Ti_3_O_18_ ceramics for the {001} and {100} planes obtained from EBSD.

**Figure 4:**
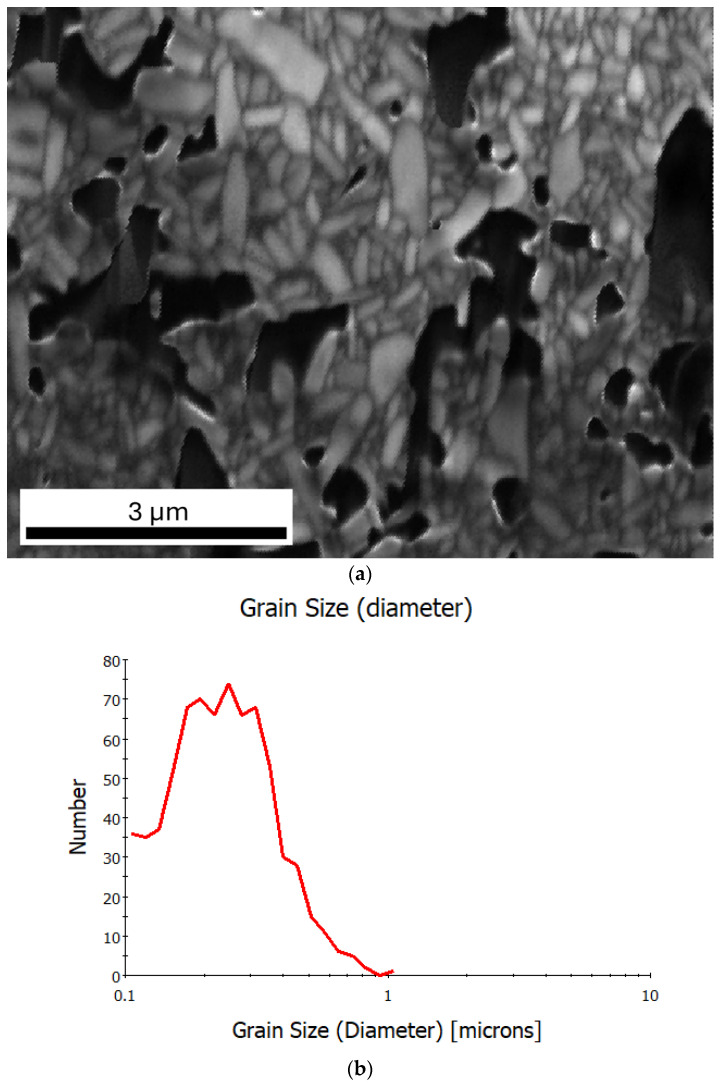
(**a**) IQ map (image quality) for Bi_6_Fe_2_Ti_3_O_18_ ceramics; (**b**) grain size distribution plot.

**Figure 5:**
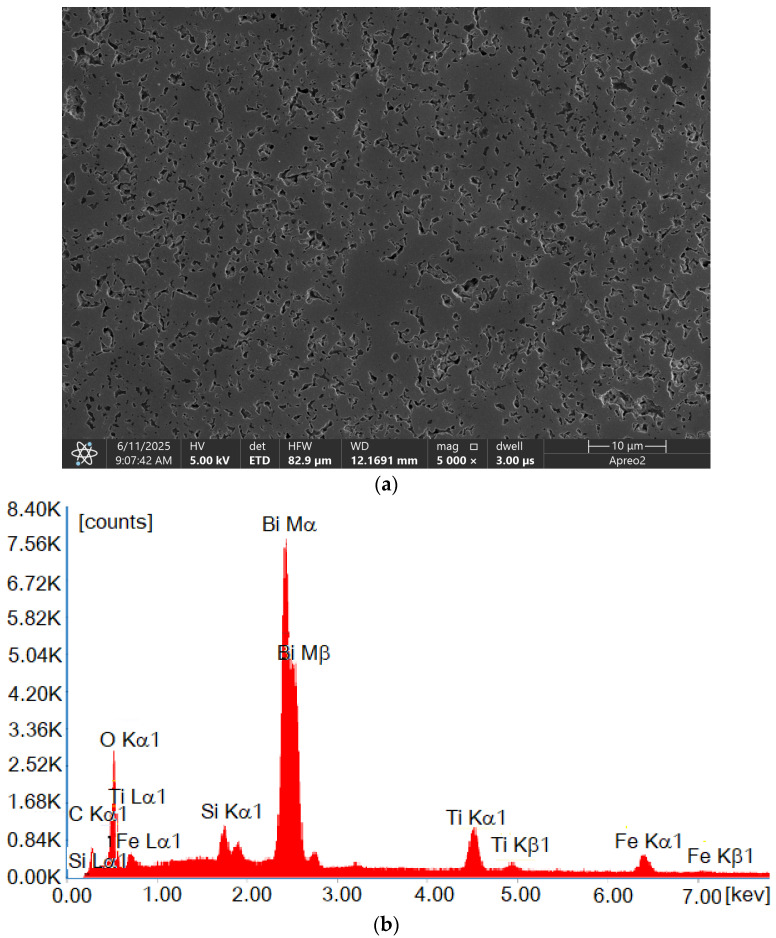
Microstructure and chemical composition of Bi_6_Fe_2_Ti_3_O_18_ ceramics obtained by using (**a**) a scanning electron microscope (secondary electron mode) and (**b**) an energy-dispersive X-ray spectrum (EDS).

Electron backscatter diffraction (EBSD), the primary tool for lattice orientation determinations, was utilized in the present study to understand the complex microstructures in the Aurivillius-type layer-structured bismuth titanium oxides. The data obtained by EBSD mapping were analyzed using the EDAX OIM Analysis™ software, and the results are shown in Figure 2, Figure 3, Figure 4 and Figure 5.

All the EBSD data analyses were performed using the EDAX OIM Analysis™.

Figure 2a presents an inverse pole figure map obtained using electron backscatter diffraction (EBSD), which reveals the crystallographic orientation in the Aurivillius-type layer-structured bismuth titanium oxides. The orientation of the grains is represented by a color-coded legend (as shown in Figure 2c), which corresponds to specific crystallographic directions according to the standard inverse pole figure key. The black regions visible in the map correspond to non-indexed areas, indicating voids within the microstructure (e.g., pores) or regions with insufficient diffraction data due to the surface quality or geometrical factors.

Figure 2b shows the orientation distribution with respect to the sample normal direction [001]. The presented distribution did not exhibit significant clustering, suggesting the absence of a strong preferred crystallographic orientation along this direction.

Further insight into the material texture is provided by the pole figures shown in Figure 3, which correspond to the {001} and {100} crystallographic planes. The color intensity represents the multiple of uniform distribution (m.u.d.), a quantitative measure indicating how much the observed orientation distribution deviates from a random texture. While there was some variation in intensity, the multiple of uniform distribution value was ~2.67, indicating the presence of a weak texture.

Image quality (IQ) maps are a grayscale representation, where the pixel intensities correspond to the quality of the Kikuchi diffraction patterns. Bright regions indicate high-quality patterns, usually obtained from well-crystallized and correctly prepared (well-polished and strain-free) surfaces, while darker regions suggest a poor diffraction quality caused by strain occurrence, crystallographic disorder, or porosity. Figure 4a presents an IQ map for the Bi_6_Fe_2_Ti_3_O_18_ ceramics. The grain boundaries are clearly visible and the black regions, in this case, correspond to pores. The microstructure shows relatively fine grains of a polycrystalline ceramic material, some with an elongated shape. This correlates with the grain size distribution plot (Figure 4b), where the grain diameter is shown in relation to the number of grains. The peaks around 0.3 to 0.4 µm indicate that most of the grains fell within this range. For sizes above 0.6 µm, the number of grains significantly decreased.

In order to complement the microstructural observations, an SEM (scanning electron microscope) image of a bigger area was introduced, as shown in Figure 5a. The observations were made using the secondary electron (SE) mode in high-vacuum mode. The presented image reveals a relatively high porosity of the Bi_6_Fe_2_Ti_3_O_18_ material. Energy-dispersive X-ray spectroscopy (EDS) measurements collected from the whole presented surface (Figure 5b) confirmed the chemical composition stability. The presence of the silicon in the spectrum can be explained by residues of silica in the pores after the polishing process. The carbon presence is explained by the sample’s surface coating.

### 3.3. Impedance Spectroscopy Measurements of Ceramics

Impedance spectroscopy (IS) is a specific technique that can be considered a subset of broadband dielectric spectroscopy (BBDS). While broadband dielectric spectroscopy covers a wide frequency range from 10^−6^ to 10^12^ Hz and includes various methods for studying the dielectric properties of materials [34], impedance spectroscopy specifically focuses on measuring the impedance of a material as a function of the frequency [20,29].

A reliable analysis of impedance spectroscopy data requires verification of the data quality and consistency. One widely accepted method for such validation is based on the Kramers–Kronig (K–K) relations, which establish a mathematical link between the real and imaginary parts of a frequency-dependent complex function. In the context of impedance, this means that the real part of the impedance spectrum, Z_Re_(ω), can be calculated from the imaginary part Z_Im_(ω), and vice versa. This property enables internal consistency checks of the measured data.

The K-K relations can be expressed in the integral form as follows [30,35]:

(1) ZRe(ω)=R∞+2π∫0∞xZIm(x)−ωZIm(ω)x2−ω2dx

(2) ZIm(ω)=2ωπ∫0∞ZRe(x)−ZRe(ω)x2−ω2dx

where R_∞_ = Z_Re_(ω→∞); *Z*_Re,i_ + j*Z*_Im,i_ represent the measured impedance at frequency ω_i_. These relations are used to confirm that the measured impedance spectrum obeys the principles of causality and linearity.

The calculations necessary for performing the K-K test were accomplished with a computer program according to the methodology described in scientific publications [30,35]. In the present study, the impedance data were found to be consistent with the K–K framework, validating the reliability of the measurements prior to further dielectric analysis.

In an ideal case, the results of impedance spectroscopy measurements over a wide range of frequencies can be presented by semicircles in a complex *Z*″-*Z*′ plane (Nyquist plot). Each semicircle represents the contribution of a particular process (electrodes and contacts, grain boundaries, and grain interior) to the total impedance of the sample.

**Figure 6:**
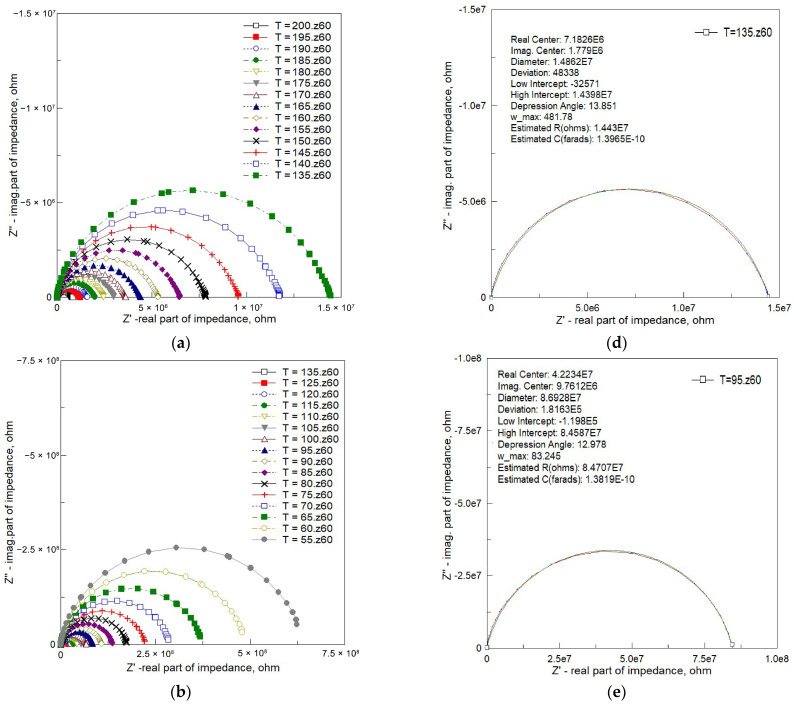

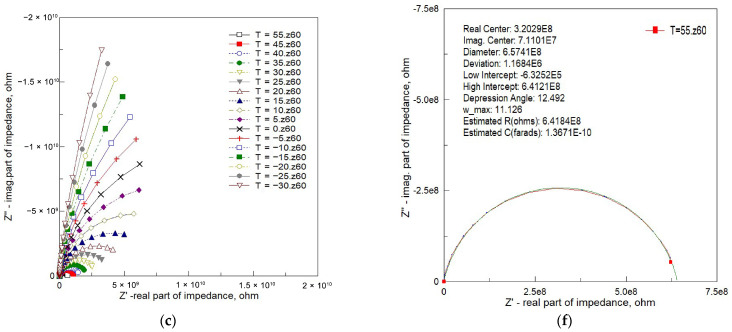
Nyquist plots of the complex impedance response for Bi_6_Fe_2_Ti_3_O_18_ ceramics. (**a**) Temperature range: 135 °C to 200 °C; (**b**) temperature range: 55 °C to 135 °C; (**c**) temperature range: −30 °C to 55 °C (linear isotropic scale); (**d**) circular arc fitting at 135 °C; (**e**) circular arc fitting at 95 °C; and (**f**) circular arc fitting at 55 °C. (Dots—experimental data; red line—line connecting experimental points; green solid lines—fitting results using “fit circle” approach.)

Nyquist plots for the Bi_6_Fe_2_Ti_3_O_18_ ceramics within the temperature range from −30 °C to +200 °C are shown in Figure 6.

One can see from Figure 6 that the measured values of the two components of complex impedance—resistance (real part, Z’) and reactance (imaginary part, Z″)—presented in the form of Nyquist plots rarely take the shape of perfect semicircles (while the isotropic scale is preserved on both axes). They are often described as depressed or deformed semicircles, with their center lying below the x-axis (Figure 6a,b). At low temperatures, the deviation from the semicircular shape is substantial (Figure 6c).

To highlight the characteristic depression of impedance semicircles, the experimental Nyquist plots were analyzed using circular arc fitting, analogous to the Cole–Cole approach for non-ideal dielectric relaxation. Figure 6d–f present fitted impedance spectra at selected temperatures. The key fitting parameters are summarized in each figure.

As the temperature decreased, the intercept of the arc on the real axis increased, indicating an increase in bulk resistance from Z_Re_ = 1.44 × 10^7^ Ω at 135 °C to Z_Re_ = 8.46 × 10^7^ Ω at 95 °C, and further to Z_Re_ = 6.41 × 10^8^ Ω at 55 °C. Concurrently, the depression angle *β* (indicative of the arc’s deviation from a perfect semicircle) and the characteristic frequency (ω_max_) decreased:

- At 135 °C: β = 13.85°, ω_max_ = 481 rad/s;
- At 95 °C: β = 12.98°, ω_max_ = 83 rad/s;
- At 55 °C: β = 12.49°, ω_max_ = 11 rad/s.

These depressed arcs are indicative of non-Debye relaxation behavior, as also suggested by the stretching exponent β extracted from the KWW fits. The centers of the fitted arcs lie below the real axis, with coordinates (in Ω) as follows:

- (7.18 × 10^6^, 1.78 × 10^6^) at 135 °C;
- (4.22 × 10^7^, 9.76 × 10^6^) at 95 °C;
- (3.20 × 10^8^, 7.11 × 10^7^) at 55 °C.

This analysis reinforces the evidence for distributed relaxation mechanisms and heterogeneity in the BFTO ceramics.

This phenomenon, called non-Debye relaxation, is attributed to the distribution of Debye relaxations with different time constants.

The Debye response is given by the following:

(3) $$Z^{*}\left(\omega \right)=Z_{\infty }+\left(Z_{S}-Z_{\infty }\right)\frac{1}{1+j\omega \tau },$$

where *Z*_s_ and *Z*_∞_ stand for the static and high-frequency-limiting values of the impedance, respectively. Equation (3) can be generalized to obtain the Cole–Cole equation [36]:

(4) $$Z^{*}\left(\omega \right)=Z_{\infty }+\left(Z_{S}-Z_{\infty }\right)\frac{1}{1+{\left(j\omega \tau \right)}^{\alpha }};\;0<\alpha \leq 1,$$

which is identical to the Debye case when α = 1, and is broader the lower the exponent α is. The Cole–Cole equation mathematically expresses the distribution of relaxations with different time constants.

**Figure 7:**
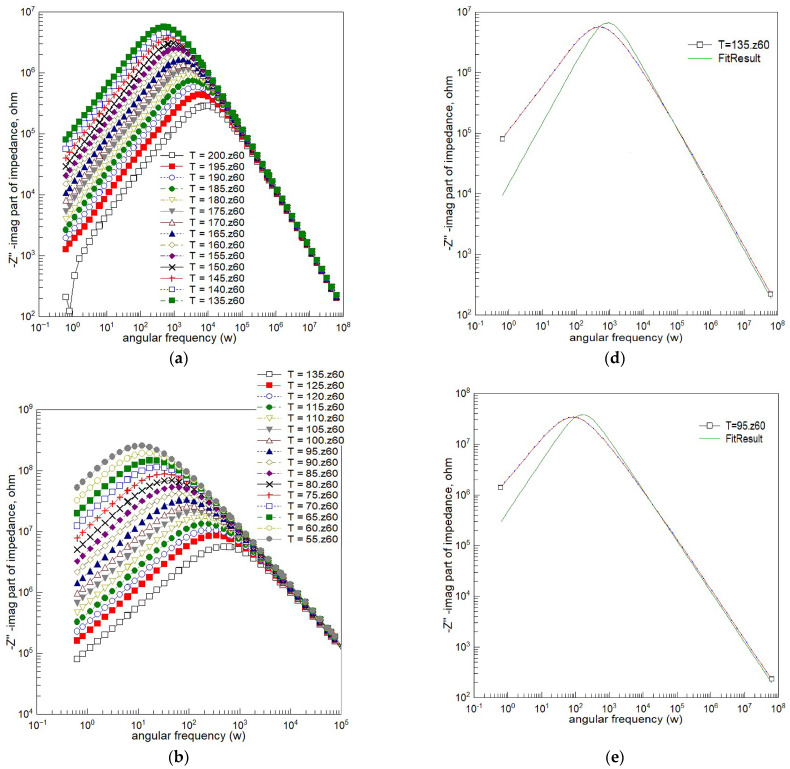

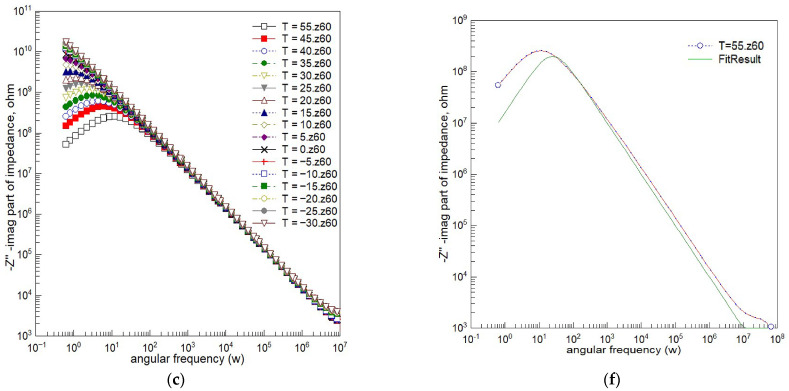
Spectroscopic plots of the imaginary component of complex impedance for Bi_6_Fe_2_Ti_3_O_18_ ceramics. (**a**) Temperature range: 135 °C to 200 °C; (**b**) temperature range: 55 °C to 135 °C; (**c**) temperature range: −30 °C to 55 °C; (**d**) Debye fitting at 135 °C; (**e**) Debye fitting at 95 °C; and (**f**) Debye fitting at 55 °C. (Dots—experimental data; red line—line connecting experimental points; green solid lines—fitting results based on Debye model.)

Spectroscopic plots of the imaginary part of complex impedance (Z″) are depicted in Figure 7. This representation is characterized by a peak, signifying the presence of dielectric relaxation in the sample. The observed peak provides insights into the dynamics and behavior of the relaxation mechanism, allowing for a more detailed analysis of the dielectric response within the material.

Figure 7 presents the imaginary part of impedance (−Z″) as a function of the angular frequency (ω) across several temperatures. The curves exhibit peaks that are noticeably broader and asymmetric compared to those predicted by the ideal Debye model, which assumes a single relaxation time. As the temperature increases, the peak frequency shifts toward higher values, reflecting thermally activated relaxation processes.

To quantify this deviation from ideality, the experimental data were fitted using a single-time-constant Debye model. The fitting results are shown for selected temperatures in Figure 7d–f. At each temperature, the experimental peaks are clearly broader and exhibit flatter tops than the corresponding Debye curves, confirming the presence of a distribution of relaxation times.

**Figure 8:**
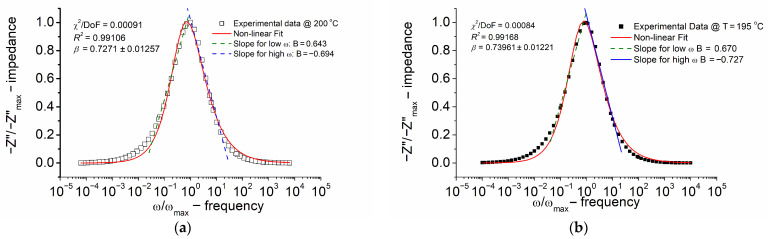

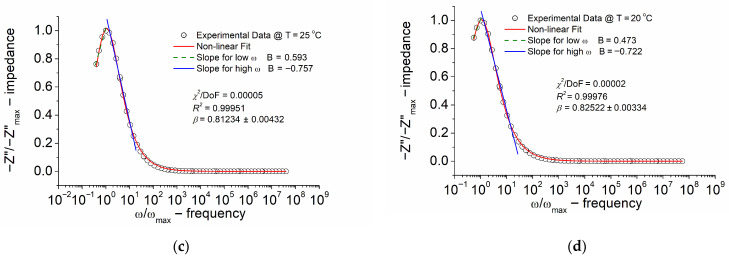
Normalized imaginary part of impedance (Z″/Z″_max_) versus normalized frequency (ω/ω_max_). (**a**) Temperature T = 200 °C; (**b**) temperature T = 195 °C; (**c**) temperature T = 25 °C; and (**d**) temperature T = 20 °C. Values of quality parameters (*χ*^2^ and *R*^2^) and the stretching parameter *β* from the KWW equation are given in the legend.

This non-Debye behavior is characteristic of complex dielectric systems, such as BFTO ceramics, where structural or compositional heterogeneities lead to a broad range of relaxation dynamics. These findings are consistent with the stretched-exponential behavior (via KWW fits, Figure 8) and with the depressed semicircular arcs in the complex impedance plane (Figure 6), both indicative of Cole–Cole-type relaxation.

A number of empirical relaxation functions have been used to describe the imaginary part of the general response function *Z*″(ω). A commonality of the functions mentioned above is that they are characterized by power laws far away from intermediate frequencies around the peak frequency ω_p_. When viewed versus a logarithmic frequency scale such as abscissa (like in Figure 7), the slope at the high- and low-frequency limits of the Debye plot is 1 and −1 at a low ω and a high ω, respectively. For the Cole–Cole equation, the slopes are α and -α at a low and high angular frequency, respectively.

To model the behavior of the imaginary component of the impedance, the alternative equation for the susceptibility functions was used [18]. A three-parameter formula for relaxation in the frequency domain given by the equation

(5) $$\frac{Z\prime \prime }{Z{\prime \prime }_{\max}}=\frac{1}{\left(1-b\right)+\frac{b}{1+b}\left[b\left(\omega_{\max}/\omega \right)+{\left(\omega /\omega_{\max}\right)}^{b}\right]};\;0<b\leq 1\;,$$

was applied to the analysis of the normalized amplitude (scaled) imaginary part of the impedance *Z*″/Z″_max_(*ω*). In Equation (5), *Z*″ represents the current value of the imaginary part of the complex impedance, Z″_max_ and *ω*_max_ define the height and position of the peak, and “*b*” is an internally independent shape parameter for high frequencies.

The KWW equation describing the relaxation function *ϕ*(*t*) [16,17] is expressed as follows:

(6) $$\phi \left(t\right)=f\exp\left[-{\left(\frac{t}{\tau }\right)}^{\beta }\right];\;0<\beta \leq 1\;,$$

where *β* is the stretching parameter, *τ* is the relaxation time, and *f* is a measure of the fraction of the experimental quantity being investigated (the amplitude parameter) that is relaxed via α-relaxation.

The relationships providing a bridge between the parameters used in the analysis of the frequency domain representation (Equation (5)) and the parameters characterizing the time-domain relaxation function (Equation (6)) are given by the following:

(7) $$b\approx \beta ,\;Z_{\max}^{\prime \prime }\approx \frac{f}{2}\beta ,\;\omega_{\max}\approx \frac{1}{\tau }\frac{1}{\sqrt{\frac{1}{\beta }\Gamma \left(\frac{1}{\beta }\right)}},$$

where *Γ* is the gamma function [37]. These relationships facilitate the translation of the findings between the time and frequency domains, aiding in understanding the relaxation behavior within the material [38].

The outcomes of modeling the normalized (in amplitude) imaginary part of impedance (Z″/Z″_max_) with a normalized frequency (ω/ω_max_) for Bi_6_Fe_2_Ti_3_O_18_ ceramics at different temperatures, carried out according to the function exhibiting the skewed shape given by Equation (5) (modified KWW formula), are depicted in Figure 8.

A visual examination of Figure 8 reveals that the experimental data align well with the model. The quality factor *R*^2^ was 0.991–0.999.

The identification of non-Debye-type relaxation phenomena was possible, as evidenced by the analysis of the stretching parameter *β* of the KWW function, within temperatures from 20 °C to 200 °C. Despite the BBDS measurements being performed for temperatures lower than 20 °C, it was found that the relaxation peak on the spectroscopic dependence of the imaginary part of impedance shifted to the lower frequency and “disappeared” from the measuring frequency window. It was found that the *β* parameter of the KWW function ranged from ~0.72 to 0.82 within the temperature range from 200 °C to 20 °C (in the impedance formalism). A lower *β* value indicates a more stretched relaxation function.

**Figure 9:**
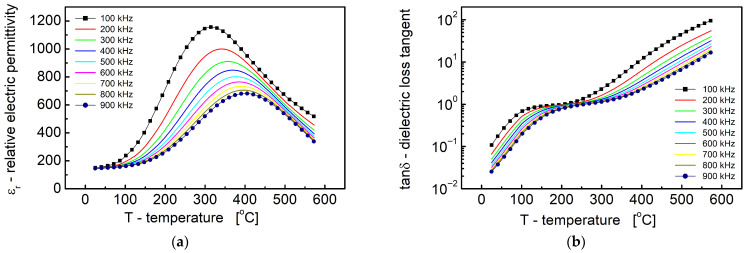
Dependence of relative dielectric permittivity (**a**) and dielectric loss tangent (**b**) on temperature.

The temperature dependence of the relative permittivity (ε_r_) and the dielectric loss tangent (tan δ) for Bi_6_Fe_2_Ti_3_O_18_ ceramics is presented in Figure 9a and Figure 9b, respectively. The experimental data were obtained over a measurement field frequency range of 100 kHz to 900 kHz. As shown in Figure 9a, the relative permittivity exhibited a distinct maximum within the temperature range of 600 K to 700 K. This peak shifted toward higher temperatures and decreased in magnitude with an increasing frequency, indicating a frequency-dispersive dielectric response. In contrast, the dielectric loss tangent (Figure 9b) initially increased slowly with temperature, reaching a value of approximately 1 between 450 K and 550 K, followed by a rapid increase at higher temperatures, exceeding a value of 5 for T > 700 K.

The experimental data were obtained over a measurement field frequency range of 100 kHz to 900 kHz. As shown in Figure 9a, the relative permittivity exhibited a distinct maximum within the temperature range of 300 °C to 400 °C. This peak shifted toward higher temperatures and decreased in magnitude with an increasing frequency, indicating a frequency-dispersive dielectric response. In contrast, the dielectric loss tangent (Figure 9b) initially increased slowly with temperature, reaching a value of approximately 1 between 100 °C and 300 °C, followed by a rapid increase at higher temperatures, exceeding a value of 5 for T > 450 °C.

This behavior is characteristic of relaxor ferroelectric materials, where diffuse phase transitions and the strong frequency dispersion of the dielectric peak are commonly observed. The shift of the ε_r_ maximum to higher temperatures with an increasing frequency suggests the presence of thermally activated dielectric relaxation processes, likely driven by local structural distortions and compositional inhomogeneities inherent to the layered Aurivillius structure of Bi_6_Fe_2_Ti_3_O_18_ [10,11].

These findings correlate well with the microstructural features revealed by the EBSD and BBDS analyses. In particular, EBSD mapping (Figure 2, Figure 3, Figure 4 and Figure 5) demonstrated significant orientation-dependent contrast and the presence of ferroelastic domains, which are known to contribute to local polarization fluctuations and dielectric dispersion. The BBDS results further confirmed the existence of domain wall activity and polar anisotropy at the sub-micron scale [20,29]. Such domain-related phenomena can give rise to a broadened dielectric response and relaxor-like behavior, as observed in the temperature-dependent permittivity measurements.

Additionally, the pronounced increase in dielectric loss (tan δ) above 700 K (Figure 7b) may be attributed to an increased electrical conductivity at elevated temperatures [13]. This is likely related to thermally activated charge carriers, such as oxygen vacancies or hopping between Fe^3+^/Fe^2+^ centers, as suggested by the defect chemistry typical of Bi-based Aurivillius oxides.

## 4. Conclusions

Bi_6_Fe_2_Ti_3_O_18_ (BFTO) ceramics were synthesized by the mixed oxide method using stoichiometric Bi_2_O_3_, TiO_2_, and Fe_2_O_3_ powders. A thermal analysis guided the optimization of the heat treatment parameters, and uniaxial hot pressing at 850 °C was employed for final densification and sintering. X-ray diffraction confirmed a tetragonal structure (space group *I4/mmm*, No. 139) for BFTO with lattice parameters of *a*_0_ = *b*_0_ = 3.8625Å, *c*_0_ = 49.585Å. The average crystallite size and rms strain was 156Å and 0.41%, respectively.

Electron backscatter diffraction revealed a random grain orientation without significant texturing, as confirmed by a pole figure analysis of the {001} and {100} planes. SEM imaging indicated a fine-grained microstructure with some porosity, which was corroborated by image quality maps. The EDS analysis verified the expected chemical composition; the carbon and silicon that were detected originated from sample preparation.

Broadband dielectric spectroscopy characterized the dielectric response over frequencies from 10 mHz to 10 MHz and temperatures from −30 °C to +200 °C. The normalized imaginary impedance (Z′/Z″_max_) spectra were modeled using the Kohlrausch–Williams–Watts (KWW) function, achieving the fitting quality (*R*^2^ = 0.991–0.999). Non-Debye relaxation was identified through the stretching parameter *β*, which ranged from approximately 0.72 to 0.82 between 200 °C and 20 °C, indicating a broad distribution of relaxation times. Below 20 °C, the relaxation peak shifted outside the measuring window.

The dielectric measurements revealed frequency-dependent maxima in the relative permittivity and a sharp increase in dielectric loss at elevated temperatures, consistent with relaxor-like ferroelectric behavior. These features align well with the structural and domain-related observations from EBSD and BBDS, which confirmed the presence of ferroelastic domains and local anisotropy.

Due to current limitations in experimental resources, techniques such as XPS and Raman spectroscopy were not included in the present study. However, we recognize their importance for investigating the electronic structure and local bonding in Aurivillius-type ceramics. Future work will focus on detailed spectroscopic studies of Bi_6_Fe_2_Ti_3_O_18_ and related Bi_4_Ti_3_O_12_–BiFeO_3_ compounds, with a comprehensive analysis to be presented in a separate publication.

## Figures and Tables

**Table 1:** Results of the qualitative X-ray diffraction phase analysis.

Chemical Formula	PDF Entry No.	Crystal Symmetry/Space Group	FoM
Bi_11_Ti_6_Fe_3_O_33_	00-054-0240	Orthorhombic/*Bm2m*, 35/*a/c* ≈ 1.0042	0.8490
Bi_5_Ti_3_FeO_15_	ICSD-74037	Orthorhombic/*Fmm2*, 42/*a/c* ≈ 1.0068	0.8204
Bi_7_Ti_3_Fe_3_O_21_	00-054-1044	Orthorhombic/*Fm2m*, 42/*a/c* ≈ 1.0044	0.8110
Bi_9_Ti_6_FeO_27_	00-042-0052	Orthorhombic/*Cmm2*, 35/*b/a* ≈ 1.015	0.7699
Bi_6_Ti_3_Fe_2_O_18_	00-021-0101	Orthorhombic ^1^/-/*b/a* ≈ 1.0018	0.7468

^1^ The space group number was not provided on the PDF card.

## Data Availability

The original contributions presented in this study are included in the article. Further inquiries can be directed to the corresponding author.
